# Knowledge about E-Cigarettes and Tobacco Harm Reduction among Public Health Residents in Europe

**DOI:** 10.3390/ijerph16122071

**Published:** 2019-06-12

**Authors:** Pietro Ferrara, Saran Shantikumar, Vítor Cabral Veríssimo, Rafael Ruiz-Montero, Cristina Masuet-Aumatell, Josep Maria Ramon-Torrell, the EuroNet MRPH Working Group on Electronic Cigarettes and Tobacco Harm Reduction

**Affiliations:** 1 Department of Experimental Medicine, University of Campania “Luigi Vanvitelli”, 80138 Naples, Italy; pietro.ferrara1@unicampania.it; 2 Warwick Medical School, University of Warwick, Coventry CV4 7AL, UK; saranshantikumar@gmail.com; 3 Public Health Unit—ACeS Lisboa Central, 1900-138 Lisbon, Portugal; vitorv@campus.ul.pt; 4 Preventive Medicine Unit, Hospital Universitario Reina Sofía/IMIBIC, 14004 Córdoba, Spain; rafaelruizmontero@gmail.com; 5 Preventive Medicine Department, Bellvitge Biomedical Research Institute (IDIBELL), University Hospital of Bellvitge, Feixa Llarga s/n, L’Hospitalet de Llobregat, 08907 Catalonia, Spain; cmasuet@bellvitgehospital.cat; 6 Clinical Science Department, School of Medicine, University of Barcelona, L’Hospitalet de Llobregat, 08907 Catalonia, Spain

**Keywords:** electronic cigarettes, harm reduction, healthcare workers, nicotine, public health, smoking cessation

## Abstract

Introduction: Although electronic cigarettes (e-cigarettes) and other tobacco-related products are becoming widely popular as alternatives to tobacco, little has been published on the knowledge of healthcare workers about their use. Thus, the aim of this study was to elicit the current knowledge and perceptions about e-cigarettes and tobacco harm reduction (THR) among medical residents in public health (MRPH). Material and Methods: A Europe-wide cross-sectional study was carried out amongst MRPH from the countries associated with the European Network of MRPH from April to October 2018 using an online questionnaire. Results: 256 MRPHs agreed to participate in the survey. Approximately half the participants were women (57.4%), with a median age of 30 years, and were mainly Italian (26.7%), Spanish (16.9%) and Portuguese (16.5%). Smoking prevalence was 12.9%. Overall, risk scores significantly differed for each investigated smoking product when compared with e-cigarettes; with tobacco cigarettes and snus perceived as more risky, and nicotine replacement therapy (NRT) and non-NRT oral medications seen as less risky (*p* < 0.01 for all). Regarding the effects of nicotine on health, the vast majority of MRPHs associated nicotine with all smoking-related diseases. Knowledge of THR was low throughout the whole sample. Conclusions: European MRPH showed a suboptimal level of knowledge about e-cigarettes and THR. Training programs for public health and preventive medicine trainees should address this gap.

## 1. Introduction

Recent data on the global tobacco epidemic published by the World Health Organization declared that, with over 1.1 billion smokers, tobacco remains the leading single preventable cause of death worldwide, killing over 7 million people each year and being responsible for one in ten deaths [1,2].

In recent years, electronic cigarettes (e-cigarettes) and the use of other tobacco-related products have become widely popular and have been extensively highlighted as alternatives to smoking or aids for smoking cessation [3,4], addressing potential challenges concerning tobacco morbidity and mortality [5].

E-cigarettes are battery-operated electronic devices that, instead of burning tobacco, produce vapors containing, amongst others, nicotine, propylene glycol and chemical additives [6,7]. Doubts remain around their safety and the United States Food and Drug Administration (FDA) warns that e-cigarettes still contain several toxic chemicals and suspected carcinogens that may be harmful to human health [8,9]. Indeed, the debate around the potential health impacts of e-cigarettes is ongoing within the scientific community, and across European countries, current policies and regulatory strategies surrounding e-cigarette use remain uncoordinated [10,11,12].

Similarly, little attention has been paid to investigating the knowledge of healthcare workers toward the use of e-cigarettes and tobacco harm reduction (THR) strategies, particularly in the public health field, although the evidence available thus far suggests a suboptimal level of awareness [13,14].

Tobacco is a public health priority and public health professionals have an important role in providing interventions for smoking cessation and prevention, both with individual therapeutic actions and from a population perspective [15]. For this reason, educational and training programs for future public health professionals should be consistent with the latest evidence-based approaches against the tobacco epidemic [14,15]. The licensing and marketing of alternative forms of smoking habits and nicotine utilization amongst users—such as e-cigarettes and THR—requires that curricular pathways for future public health professionals have to parallel the epidemiological transition of smokers’ behaviors, in order to better tackle the tobacco threat and promote healthy lifestyles. In this frame, it has been also recognized that smokers who want to quit or shift to other nicotine products are often poorly helped by public health authorities [16,17].

THR refers to a public health strategy that aims to reduce the health risks associated with tobacco smoking. Since the 1970s, when the late psychiatrist Russell famously stated that, “*smokers smoke for the nicotine, but die from the tar*”, THR has been a long-standing issue. Accumulating evidence suggests that safer nicotine alternatives may garner considerable public health benefits and reduce the morbidity and mortality caused by smoking [18,19,20]. As early as 2003, the WHO Framework Convention on Tobacco Control acknowledged the utility of this strategy by including THR in tobacco control policies, due to its important role in eliminating or reducing population consumption of tobacco products and exposure to tobacco smoke [17,21]. Moreover, results from practical experience have suggested an emerging optimism (for instance, with the general public preference for e-cigarettes worldwide, and snus use in Sweden), despite divided opinions [22]. Yet, 40 years from the conception of THR, it still raises many controversial questions, mainly due to inconclusive findings regarding the actual potential danger of THR tools and devices and the need for further prospective clinical research in order to highlight the safety and addictive potential of these products [23].

Therefore, the aim of this study was to evaluate, through a European cross-sectional survey, the current level of knowledge about e-cigarettes and THR strategies amongst medical residents/registrars in public health (MRPH), assessing their knowledge level in the issues of interest and addressing the need to make fundamental changes in education and training programs, based on the best evidence available.

## 2. Materials and Methods

### 2.1. Population and Sample

The target population of our research—MRPH—corresponds to health professionals (including non-medical healthcare personnel) enrolled in post-graduate training in public health. Across European countries, education programs for MRPH differ by curricular pathway, structure, as well as by training institutions [24]. Indeed, MRPH can be employed in university departments, hospitals, epidemiology agencies, and other healthcare institutions. As a consequence of this heterogeneity, the career roles and responsibilities of MRPH include a vast variety of interventions to reduce risk factors and the social burden of diseases such as health promotion, health communication, policy and management, preventive medicine, and epidemiological methods.

For the purposes of this study, an online cross-sectional survey was conducted between April and October 2018 in a sample of MRPH from the member countries of the European Network of Medical Residents in Public Health (EuroNet MRPH). The countries associated with EuroNet MRPH during the study period were Bosnia, Croatia, France, Ireland, Italy, Malta, Moldova, the Netherlands, Poland, Portugal, Slovenia, Spain, Turkey, and the United Kingdom.

The EuroNet MRPH Board was informed about the nature and protocol of the study. Approval to carry out this research was granted by the EuroNet MRPH General Assembly and a Working Group on Electronic Cigarettes and Tobacco Harm Reduction was subsequently formed.

Due to the diversity of the MRPH population in terms of numbers and employment settings across European countries, it was difficult to identify sample units and gain access to them. For this reason, we chose a snowball sampling procedure to recruit interview participants, since this method is recognized as useful for reaching cases in a hard-to-reach uniquely defined network and, according to literature, it can be utilized to make statistical inferences for examining relationships present in the target population [25,26,27]. Specifically, we firstly delivered the self-administered anonymous questionnaire to all EuroNet MRPH National Commissions, individual members, and national MRPH associations, where these existed. Each of these were then invited to recruit MRPH, using existing networks or other means, to participate in the survey. The survey was also uploaded to these associations’ social media networks.

### 2.2. Survey Instrument

Data were collected using a self-administered semi-structured online survey, designed at the primary centre of this investigation (IDIBELL, Preventive Medicine Department, Barcelona, Spain), in accordance with the literature [14].

The survey was divided into three sections. The first section captured demographic and professional characteristics of the MRPH, including age, sex, country of residency, residency year, practice setting, and attendance at any specific training in smoking cessation during residency. The second section assessed smoking status and habits of the respondents. They were asked to indicate whether they were current, former or non-smokers. Respondents who were smokers could choose the smoking product they used (for instance, cigarettes, e-cigarettes, or others). Cigarette smokers were invited to indicate the number of cigarettes smoked per day and if they were considering quitting. The third section consisted of 26 items that aimed to assess MRPHs’ knowledge about and attitudes toward e-cigarettes (i.e., electronic nicotine delivery systems, including heat-not-burn products) and THR. Attitudes were measured through Likert-type scales, ranging from 1 (low risk) to 10 (high risk), and stratified into two categories for scoring the perceived health risk of ten smoking products (tobacco cigarettes, snus, e-cigarettes, nicotine replacement therapy [NRT], non-NRT oral medications) and components (nicotine, inhaled smoke, carbon monoxide, tobacco, tobacco residue). The perceived contribution of nicotine to four smoking-related disease outcomes (i.e., smoking-related diseases in general, lung cancer, cancer in other organs, and atherosclerosis) was assessed as one of “extremely important”, “very important“, “important”, “unimportant”, and “no contribution”; 20 other items were used to further investigate MRPHs’ thoughts and knowledge using a series of statements (with *yes/no/do not know* or *higher/equal/lower/do not know* responses). The questions used in the survey and response options are reported in File S1 (Supplementary Materials).

The questionnaire was delivered to all participants via professional online survey software (Google^®^ Forms, Google LLC, Mountain View, CA, USA) and was launched in English. All MRPH received an email inviting them to complete the survey accessible via an embedded Uniform Resource Locator (URL) link. Clear preliminary statements provided information about the study, and participants provided informed consent prior to completing the survey. Involvement was voluntary and no incentives were offered. MRPH were also informed that all information gathered would be anonymous and that confidentiality would be maintained by omitting personal identifying information from the analysis.

### 2.3. Content Validity

Once the survey was delivered to EuroNet MRPH, the Working Group on E-Cigarettes evaluated the overall acceptability of the questionnaire in terms of length, clarity, and question formats. Based on its suggestions, minor revisions were made to include changes to questionnaire item wording and format.

After collection, data were automatically stored in an electronic spreadsheet and were cleaned in order to reduce the risk of measurement error. Cronbach’s coefficient alpha (*α*) was measured to gauge the internal consistency of questions that loaded onto the same factors (i.e., questions related to the contribution of nicotine to diseases (a), and health risk scores for smoking products and components (b)). Construct validity was also explored using principal component analysis (PCA), reported in File S2 (Supplementary Materials).

### 2.4. Statistical Analysis

The statistical analysis consisted of descriptive and inferential analyses using non-parametric tests. Non-normal distributions of the underlying data were confirmed using the Kolmogorov-Smirnov test (*p* < 0.05 for all). Continuous variables were expressed as median and range; categorical variables were described as number and percentage. Mann-Whitney U tests (2 groups) or Kruskal-Wallis H tests (>2 groups) were used to assess differences between medians, and chi-square (*χ*^2^) or Fisher’s exact tests were used to assess differences between categorical variables. All statistical tests were two-tailed and differences were considered to be statistically significant where *p* ≤ 0.05. Data were analysed using Stata v. 10 (Stata Statistical Software: Release 10; StataCorp LP: College Station, TX, USA).

## 3. Results

Overall, 257 MRPH opened the survey link, of which only one did not agree to participate. Respondents’ socio-professional characteristics are presented in Table 1.

Approximately half the participants were female (57.4%), with a median age of 30 years, and were mainly Italian (26.7%), Spanish (16.9%) and Portuguese (16.5%). The most frequent employment and training setting for MRPH respondents was universities (40.5%), with another 27.8% in hospitals, 14.7% in public health institutes, and 6.7% in the primary care sector. Only 51 (20.1%) MRPH had attended specific training in smoking cessation during their residency so far. Regarding respondents’ smoking status and habits, 33 (12.9%) self-reported smoking on a regular basis. Cigarettes were the most commonly used product (69.7%), whereas only 7 (21.2%) current smokers declared they used e-cigarettes. Overall, one third (33.3%) of current and former smokers came from Italy.

**Figure 1:**
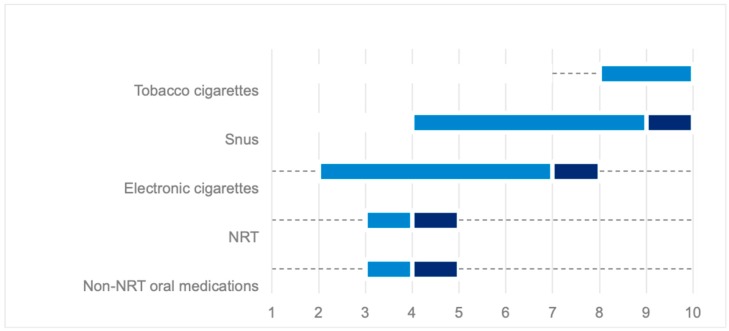
Health risk scores for smoking products. NRT, nicotine replacement therapy.

**Figure 2:**
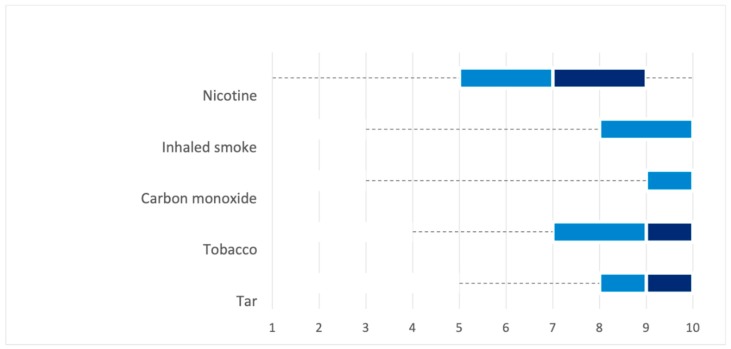
Health risk scores for smoking components. Tar, tobacco residue.

MRPH were asked to score the perceived risk to health of smoking products and components on a 10-point Likert-type scale ranking from 1 (low risk) to 10 (high risk). Boxplots in Figure 1 and Figure 2, and Table S1 (Supplementary Materials, File S3) show the results.

Compared with tobacco cigarettes, the perceived risk to health scores of snus and e-cigarettes were significantly lower (*p* < 0.001). In addition, risk scores significantly differed for each investigated smoking product when compared with e-cigarettes, with tobacco cigarettes and snus perceived as riskier, and NRT and non-NRT oral medications seen as less risky (*p* < 0.01 for all). Interestingly, no significant differences in perceived health risk scores for smoking products and components were found between smokers (current and former) and non-smokers, except for e-cigarettes with smokers declaring a lower risk (Tables S2 and S3, Supplementary Materials, File S3). Country-based differences in scoring the risk of smoking products and components were found (Table S4, Supplementary Materials, File S3).

**Figure 3:**
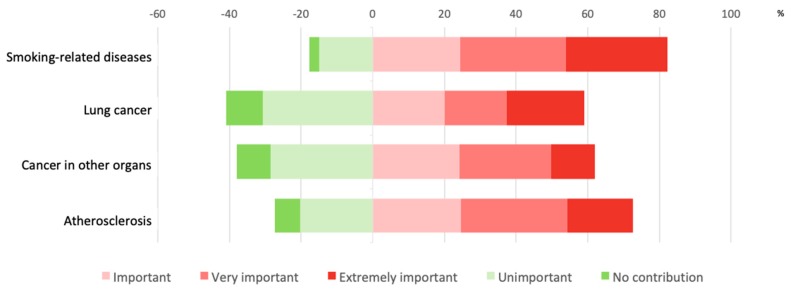
Stacked bar-charts indicating MRPHs’ responses on the contribution of nicotine to diseases.

Respondents also reported how important they believed the contribution of nicotine is to some diseases. Stacked bar-charts in Figure 3 and Table S5 (Supplementary Materials, File S3) highlight that the vast majority of respondents believed nicotine to be a significant contributor toward disease, with 82.2% associating nicotine with all smoking-related diseases, 59.1% indicating an important contribution to lung cancer, 62.1% to cancer in other organs, and 72.7% considering it responsible for atherosclerosis.

MRPHs’ responses on e-cigarettes and THR are presented in Table 2. Regarding e-cigarettes, half the MRPH considered their health risk to be lower than that of tobacco cigarettes (53.1%), but that they had an equal dependence potential (51.4%). The vast majority (84.9%) agreed that e-cigarettes could generate addiction.

Only 31.7% respondents felt that e-cigarettes were effective devices for smoking cessation, with a similar proportion (36.4%) agreeing that concomitant use of e-cigarettes and tobacco effectively reduces the number of smoked cigarettes. On this point, a large proportion of respondents stated that they would not recommend e-cigarettes to patients, as either a smoking cessation aid (63.9%) or as a strategy for reducing the number of smoked cigarettes (46.6%).

Regarding THR strategies, around three-quarters of MRPH admitted to not having heard about modified-risk tobacco products and not being able to compare their health risk to smoking and e-cigarettes.

Tables S6–S8 (Supplementary Materials, File S3) show differences in these items by respondents’ smoking status, previous attendance at specific training in smoking cessation, and residency characteristics.

Internal reliability estimates of the survey suggested a high degree of internal consistency for the investigated items (Cronbach’s *α* = 0.79 (a) and 0.75 (b)).

## 4. Discussion

To our knowledge this is the first Europe-wide study investigating the awareness of healthcare professionals about e-cigarettes and THR strategies, yielding important findings on the current level of knowledge amongst a sample of MRPH from countries associated with EuroNet MRPH.

The first interesting finding is the prevalence of smokers in our MRPH respondents, with 12.9% self-reporting regular use of cigarettes and other tobacco products, and a further 9.4% being former smokers. Our results displayed a considerably lower rate of self-reported smokers compared with the European adult population (28%) [28], suggesting the potential influence of a medical background on smoking habits. However, the observed percentage was much higher than that documented in physicians in the United States [29]. On this point, accumulating evidence has shown that smoking trends may not be uniform across different countries, with healthcare workers in some regions still smoking at fairly high rates, especially in the European context [29] where the highest prevalence of daily smokers was observed [28]. Overall, the proportion of healthcare professionals who smoke varies within contexts, also depending on local policies, education and training background, and risk factors [30]. Similarly, with specific reference to the prevalence of tobacco use among medical residents/registrars, country-based differences have also been observed, with a Spanish study by Sánchez et al. [30] finding a prevalence of current and former smokers, respectively, of 6.5% and 5.2%, while a multicenter survey of MRPH in Italy revealed a smoking prevalence of 20.9% [15].

On the whole, respondents perceived a moderate/high health risk for nicotine, also considering its impact on smoking-related diseases, including cancer and atherosclerosis. However, they agreed in ranking NRT risk as lower than that of smoking. It is known that nicotine is a highly addictive substance, being the most common addiction to a legal drug worldwide.

The overestimation of the harmful effects of nicotine in humans is a widespread and pervasive belief amongst healthcare workers, perhaps due to the opinion that publicly minimizing the risk potential of nicotine might convey a false underestimation of smoking-related health risks [14]. Nevertheless, currently available evidence does not suggest that nicotine promotes cancer pathway activation, and its contribution to cardiovascular disease is lower than that of tobacco smoke [31].

Concerning e-cigarettes, the majority of MRPH considered their health risk and dependence potential equal to, or lower than, that of tobacco cigarettes, in accordance with general population beliefs [32], but only one-third ranked them as effective for smoking reduction or cessation. Indeed, the published literature currently available seems to be insufficient for reliably drawing firm conclusions, or creating a consensus, on the effectiveness of e-cigarettes as a smoking cessation or harm reduction tool [4,33,34]. Pertinently, a recently published trial that compared e-cigarettes and NRT in adults seeking help to quit smoking found that the former showed superiority in one-year abstinence rates [35].

Again, the majority of the surveyed MRPH correctly stated that some alternative nicotine products (e-cigarettes, snus, NRT, etc.) were safer than smoking cigarettes. These data are also consistent with users’ beliefs: a recently published nationally representative survey of U.S. adults found that, in the general population e-cigarettes were seen as less harmful compared to cigarettes, even though the perceptions of harmfulness varied between the other non-cigarette-tobacco products [36].

Three-quarters of MRPH admitted to never having heard about THR during their medical training or public health residency. Nevertheless, e-cigarettes, heat-not-burn tobacco devices, snus, and modified-risk nicotine-containing products continue to attract consumers. For this reason, a paradigm shift is required in public health sectors, with professionals reappraising THR itself and acknowledging that it is not risk-free [20].

It is worth underlining that only one-third of respondents considered e-cigarettes an effective device for smoking cessation and the same proportion agreed with the idea that electronic devices should be recommended to patients both as a smoking cessation aid and as a tool for reducing the number of cigarettes smoked.

Briefly, certain sensitivities toward tobacco control strategies are commonly traceable among the sampled MRPH, even if respondents’ general opinions on e-cigarettes and THR were discordant, likely because they might have not received adequate training on tobacco and smoking during their residency. In fact, only 20.1% of participants attended specific training and this attendance was not found to be associated with improved knowledge of these topics amongst MRPH.

Overall, our findings suggest a need for educative interventions in public health residency programs in Europe. We found that MRPHs are not optimally confident with smoking control strategies, despite being aware of the importance of their role as physicians in anti-tobacco campaigns.

In this respect, public health stakeholders in Europe are promoting a greater awareness of e-cigarettes and their risks. The present European regulation for electronic nicotine delivery systems requires that advertising and promotion rules for tobacco products also apply to e-cigarettes, with packaging that should provide information on toxicity and addictiveness, health warnings, and the list of all the substances contained—including the exact level of nicotine (which should be at a concentration level of no more than 20 mg/mL) [37,38]. However, not all European countries are currently complying with this Tobacco Products Directive. The European Public Health Association recently published a position statement on the state of the evidence, supporting a precautionary approach to the relative safety of e-cigarettes compared with traditional cigarettes, and promoting stronger dissemination of knowledge amongst public health professionals [38].

It is known that assessing medial trainees’ knowledge is a validated method for tailoring educational programs to the actual training needs of medical professionals [39,40]; this has particular importance regarding smoking-related topics due to their constant changes. Thus, our study calls attention to the need for a revision of educational portfolios for European medical trainees in public health and preventive medicine, introducing “real life” issues, such as smoking/harm reduction and cessation concepts.

Some potential limitations must be considered in interpreting the findings of this survey. Firstly, the cross-sectional nature does not allow us to prospectively determine a causal effect of the detected items. Secondly, possible non-response bias must be considered, since we were unable to assess the characteristics of MRPH who chose to not participate in the survey. Indeed, the EuroNet Working Group was unable to determine the total number of the whole European MRPH population, thus a response rate could not be calculated. For this reason, it is possible that the findings of our study may not be representative of all European MRPH, and we would strongly encourage further studies investigating this topic of interest. Thirdly, launching the questionnaire in English might have led some MRPH from non-English speaking countries to not answer. However, healthcare professionals, particularly those employed in public health sectors, are expected to speak English. Lastly, whilst the data revealed some negative perceptions toward e-cigarettes and THR, the responses did not allow for an exploration of context-related associations with correct/incorrect knowledge or perceptions (for instance, country-based programs), or for their likely multifactorial nature. Further research should address these issues.

Despite these limitations, we believe that the aims of this research have been achieved, by identifying novel information on MRPHs’ perceptions toward e-cigarettes and THR, and highlighting the need for further studies to better describe determinant factors for future public health professionals’ preparedness in facing challenges that could emerge in their future clinical practice.

## 5. Conclusions

European MRPH showed a suboptimal level of knowledge about e-cigarettes and THR. This has important implications, since policymakers and stakeholders should address this gap and re-design relevant training programs for public health and preventive medicine trainees.

## Figures and Tables

**Table 1:** Baseline characteristics of the study population.

Baseline Characteristics (*n* = 256)	
Characteristic *	Median (Range) or *n* (%)
Sex	
Male	106 (42.6)
Female	143 (57.4)
Age (years)	30 (23–53)
Country of residency	
Croatia	5 (1.9)
France	18 (7.1)
Italy	68 (26.7)
Portugal	42 (16.5)
Slovenia	5 (1.9)
Spain	43 (16.9)
United Kingdom	32 (12.5)
Others	42 (16.5)
Residency year	
1st	76 (31.3)
2nd	51 (21.0)
3rd	43 (17.7)
4th	53 (21.8)
5th	9 (3.7)
6th	11 (4.5)
Setting of practice	
University	102 (40.5)
Hospital	70 (27.8)
Primary care	17 (6.7)
Public health Institute/Agency	37 (14.7)
Other Health Facilities	26 (10.3)
Attended specific training in smoking cessation during residency	
Yes	51 (20.1)
No	203 (79.9)
**Smoking status and habits (*n* = 33)**	
Smokers	
Yes	33 (12.9)
No	198 (77.7)
Former smoker	24 (9.4)
Smoked products ^§^	
Cigarettes	23 (69.7)
Number of smoked cigarettes per day	7 (2–25)
E-cigarettes	7 (21.2)
Roll-your-own tobacco	8 (24.2)
Others	7 (21.2)
Considering quitting	
Yes	23 (69.7)
No	10 (30.3)

* Number for each item may not add up to total number of study population due to missing values; ^§^ Interviewees could choose more than one item.

**Table 2:** Participants’ responses on e-cigarettes and tobacco harm reduction.

Questions *	*n* (%)
The health risk of nicotine replacement therapies compared to smoking is:	
Higher	4 (1.6)
Equal	16 (6.3)
Lower	223 (88.1)
Do not know	10 (4.0)
The health risk of electronic cigarettes compared to smoking is:	
Higher	10 (4.0)
Equal	74 (29.4)
Lower	149 (53.1)
Do not know	19 (7.5)
The harmful effect of electronic cigarettes is due to the diethylene glycol:	
Yes	57 (22.5)
No	28 (11.1)
Do not know	168 (66.4)
Electronic cigarettes can generate addiction:	
Yes	214 (84.9)
No	10 (4.0)
Do not know	28 (11.1)
The dependence potential of electronic cigarettes compared to smoking is:	
Higher	26 (10.3)
Equal	130 (51.4)
Lower	65 (25.7)
Do not know	32 (12.6)
Electronic cigarettes are more expensive than normal tobacco:	
Yes	83 (32.8)
No	81 (32.0)
Do not know	89 (35.2)
Electronic cigarettes are safer than tobacco:	
Yes	111 (44.1)
No	95 (37.7)
Do not know	46 (18.2)
Electronic cigarettes are effective devices for smoking cessation:	
Yes	80 (31.7)
No	131 (52.0)
Do not know	41 (16.3)
As a Public Health professional, would you recommend the electronic cigarette as smoking cessation aid to a patient?	
Yes	69 (27.4)
No	161 (63.9)
Do not know	22 (8.7)
As a Public Health professional, would you recommend the electronic cigarette to a patient for reducing the number of smoked cigarettes?	
Yes	110 (43.5)
No	118 (46.6)
Do not know	25 (9.9)
Do you think that the concomitant use of electronic cigarettes and tobacco will effectively reduce the number of smoked cigarettes?	
Yes	92 (36.4)
No	115 (45.4)
Do not know	46 (18.2)
Do you think that medical community and healthcare workers should take a position in favor of the electronic cigarettes?	
Yes	73 (29.0)
No	130 (51.6)
Do not know	49 (19.4)
Do you think that electronic cigarettes should be prohibited?	
Yes	66 (26.1)
No	143 (56.5)
Do not know	44 (17.4)
Have you heard of modified-risk tobacco?	
Yes	62 (24.5)
No	191 (75.5)
The health risk of modified-risk tobacco products compared to smoking is:	
Higher	6 (2.4)
Equal	29 (11.6)
Lower	33 (13.3)
Do not know	181 (72.7)
The health risk of modified-risk tobacco products compared to electronic cigarettes is:	
Higher	23 (9.2)
Equal	25 (10.0)
Lower	8 (3.2)
Do not know	194 (77.6)
As a Public Health professional, would you recommend modified-risk tobacco products to reduce tobacco-related problems?	
Yes	14 (5.6)
No	59 (23.6)
Do not know	177 (70.8)

* Number for each item may not add up to total number of study population due to missing values.

## Supplementary Materials

The following are available online at https://www.mdpi.com/1660-4601/16/12/2071/s1, File S1: Survey. Questions used in the survey; File S2: Results of Principal Component Analysis. Principal Component Analysis and Scree-Plot of the Eigenvalues Performed to Explore the Construct Validity of the Survey; File S3: Supplementary Tables.

## Abbreviations

E-cigarette, electronic cigarette; EuroNet MRPH, European Network of Medical Residents in Public Health; MRPHs, medical residents in public health; THR, tobacco harm reduction; NRT, nicotine replacement therapy; TAR, tobacco residue.
