# Supplementary material for: Knowledge about E-Cigarettes and Tobacco Harm Reduction among Public Health Residents in Europe
Source: Int J Environ Res Public Health. 2019 Jun 12;16(12):2071. doi: 10.3390/ijerph16122071 (PMC6617304; doi:10.3390/ijerph16122071)
Supplement: Supplementary file 1 [file ijerph-16-02071-s001.zip › ijerph-510448-supplementary/ijerph-510448-supplementary.pdf]

Knowledge towards e-cigarettes and harm reduction among Public Health Residents in Europe

File 1
SURVEY

A1. *Participant characteristics*

1. **Gender:** □ M □ F
2. **Year of birth:** *in years*
3. **Country of residency:** [Bosnia / Croatia / France / Ireland / Italy / Malta / Moldova / The Netherlands / Poland / Portugal / Slovenia / Spain / United Kingdom / Other, *specify*:…
4. **Residency year:** □ 1 □ 2 □ 3 □ 4 □ 5 □ 6
5. **Setting of practice:** □ University □ Hospital □ Other facilities: *indicate*…
6. **Have you attended specific training in smoking cessation during residency**:
□ Yes □ No

A2. *Smoking status and habit*

7. **Current smoker:** □ Yes □ No □ Former smoker
    7.A - ***If current smoker, which of the following tobacco products do you use?***
    □ Cigarettes □ Electronic Cigarettes □ Other*: *indicate*…
    *(including pipe tobacco, waterpipe tobacco, roll-your-own tobacco, cigars, chewing tobacco, nasal tobacco, tobacco for oral use, or other than the listed tobacco related products)
    7.B - ***If cigarettes smoker:*** *indicate the number per day*
    7.C - ***If current smoker, are* you considering quitting:** □ Yes □ No

B. *Knowledge and attitudes*

Using the scale below from 1 (low risk) to 10 (high risk), please indicate the risk score for health of the following products and smoking components

8. **Health risk score for products:**

Tobacco cigarettes:	(low risk) □ 1 □ 2 □ 3 □ 4 □ 5 □ 6 □ 7 □ 8 □ 9 □ 10 (high risk)
Snus:	(low risk) □ 1 □ 2 □ 3 □ 4 □ 5 □ 6 □ 7 □ 8 □ 9 □ 10 (high risk)
Electronic cigarettes:	(low risk) □ 1 □ 2 □ 3 □ 4 □ 5 □ 6 □ 7 □ 8 □ 9 □ 10 (high risk)
Nicotine replacement therapy (NRT):	(low risk) □ 1 □ 2 □ 3 □ 4 □ 5 □ 6 □ 7 □ 8 □ 9 □ 10 (high risk)
Non-NRT oral medications:	(low risk) □ 1 □ 2 □ 3 □ 4 □ 5 □ 6 □ 7 □ 8 □ 9 □ 10 (high risk)

9. **Health risk score for smoking components:**

Nicotine:	(low risk) □ 1 □ 2 □ 3 □ 4 □ 5 □ 6 □ 7 □ 8 □ 9 □ 10 (high risk)
Inhaled smoke:	(low risk) □ 1 □ 2 □ 3 □ 4 □ 5 □ 6 □ 7 □ 8 □ 9 □ 10 (high risk)
Carbon monoxide:	(low risk) □ 1 □ 2 □ 3 □ 4 □ 5 □ 6 □ 7 □ 8 □ 9 □ 10 (high risk)
Tar:	(low risk) □ 1 □ 2 □ 3 □ 4 □ 5 □ 6 □ 7 □ 8 □ 9 □ 10 (high risk)
Tobacco:	(low risk) □ 1 □ 2 □ 3 □ 4 □ 5 □ 6 □ 7 □ 8 □ 9 □ 10 (high risk)

Using the scale below, please indicate how important you believe each of the following statements to be

10. **Contribution of nicotine to smoking-related diseases:**
□ Extremely important □ Very important □ Important
□ Unimportant □ No contribution

11. **Contribution of nicotine to lung cancer:**
□ Extremely important □ Very important □ Important
□ Unimportant □ No contribution

12. **Contribution of nicotine to cancer in other organs:**
□ Extremely important □ Very important □ Important
□ Unimportant □ No contribution

13. **Contribution of nicotine to atherosclerosis:**
□ Extremely important □ Very important □ Important
□ Unimportant □ No contribution

You will now be asked about your thoughts and knowledge on a series of statements

14. **The health risk of nicotine replacement therapies compared to smoking is:**
□ Higher □ Equal □ Lower □ Do not know

15. **The health risk of electronic cigarettes compared to smoking is:**
□ Higher □ Equal □ Lower □ Do not know

16. **The harmful effect of electronic cigarettes is due to the diethylene glycol:**
□ Yes □ No □ Do not know

17. **Electronic cigarettes can generate addiction:**
□ Yes □ No □ Do not know

18. **The dependence potential of electronic cigarettes compared to smoking is:**
□ Higher □ Equal □ Lower □ Do not know

19. **Electronic cigarettes are more expensive than normal tobacco:**
□ Yes □ No □ Do not know

Knowledge towards e-cigarettes and harm reduction among Public Health Residents in Europe

20. **Electronic cigarettes are safer than tobacco:**
□ Yes □ No □ Do not know

21. **Electronic cigarettes are effective devices for smoking cessation:**
□ Yes □ No □ Do not know

22. **As a Public Health professional, would you recommend the electronic cigarette as smoking cessation aid to a patient?**
□ Yes □ No □ Do not know

23. **As a Public Health professional, would you recommend the electronic cigarette to a patient for reducing the number of smoked cigarettes?**
□ Yes □ No □ Do not know

24. **Do you think that the concomitant use of electronic cigarettes and tobacco will effectively reduce the number of smoked cigarettes?**
□ Yes □ No □ Do not know

25. **Do you think that medical community and healthcare workers should take a position in favour of the electronic cigarettes?**
□ Yes □ No □ Do not know

26. **Do you think that electronic cigarettes should be prohibited?**
□ Yes □ No □ Do not know

27. **Have you heard of modified-risk tobacco?**
□ Yes □ No

28. **The health risk of modified-risk tobacco products compared to smoking is:**
□ Higher □ Equal □ Lower □ Do not know

29. **The health risk of modified-risk tobacco products compared to electronic cigarettes is:**
□ Higher □ Equal □ Lower □ Do not know

30. **As a Public Health professional, would you recommend modified-risk tobacco products to reduce tobacco-related problems?**
□ Yes □ No □ Do not know

Knowledge towards e-cigarettes and harm reduction among Public Health Residents in Europe

File 2

RESULTS OF PRINCIPAL COMPONENTS ANALYSIS

In order to check content validity of the survey instrument designed for the purposes of the study, Principal Components Analysis (PCA) were conducted on variables that point back to the health risk scores for smoking products and components:

1. *Tobacco cigarettes*
2. *Electronic cigarettes*
3. *Snus*
4. *Nicotine replacement therapy (NRT)*
5. *Non-NRT oral medications*
6. *Nicotine*
7. *Inhaled smoke*
8. *Carbon monoxide*
9. *Tobacco*
10. *Tobacco residue*

Results are presented in Table PCA 1 and Figure PCA 1.

Table PCA 1. Results of Principal Components Analysis and Factor Analysis.

Eigenvalues of the principal components				
	**Eigenvalue**	**Difference**	**Proportion of variance explained**	**Cumulative variance explained**
1	3.22	1.31	0.32	0.32
2	1.91	0.85	0.19	0.51
3	1.06	0.17	0.11	0.62
4	0.89	0.19	0.09	0.71
5	0.70	0.12	0.07	0.78
6	0.58	0.03	0.06	0.84
7	0.55	0.09	0.05	0.89
8	0.46	0.08	0.05	0.94
9	0.38	0.12	0.03	0.97
10	0.26	-	0.03	1.00

Factor loadings			
Variables	Factor1	Factor2	Factor3
1	0.30	0.31	- 0.19
2	0.39	- 0.12	- 0.20
3	0.39	- 0.07	- 0.16
4	0.30	- 0.48	0.29
5	0.26	- 0.44	0.43
6	0.35	- 0.25	- 0.21
7	0.31	0.27	- 0.09
8	0.34	0.18	- 0.29
9	0.28	0.36	0.35
10	0.18	0.41	0.60

Eigenvalue represents the variance of the component and is considerate to be critical for values > 1. All the values add to the sum of the number of the variables. The *Difference* is the size of a component's eigenvalue and the next component's eigenvalue. Thus, the first three principal components with eigenvalues > 1 explained the 67% of the information in the data set.

Factor loadings reflected the multiplicity of the survey.

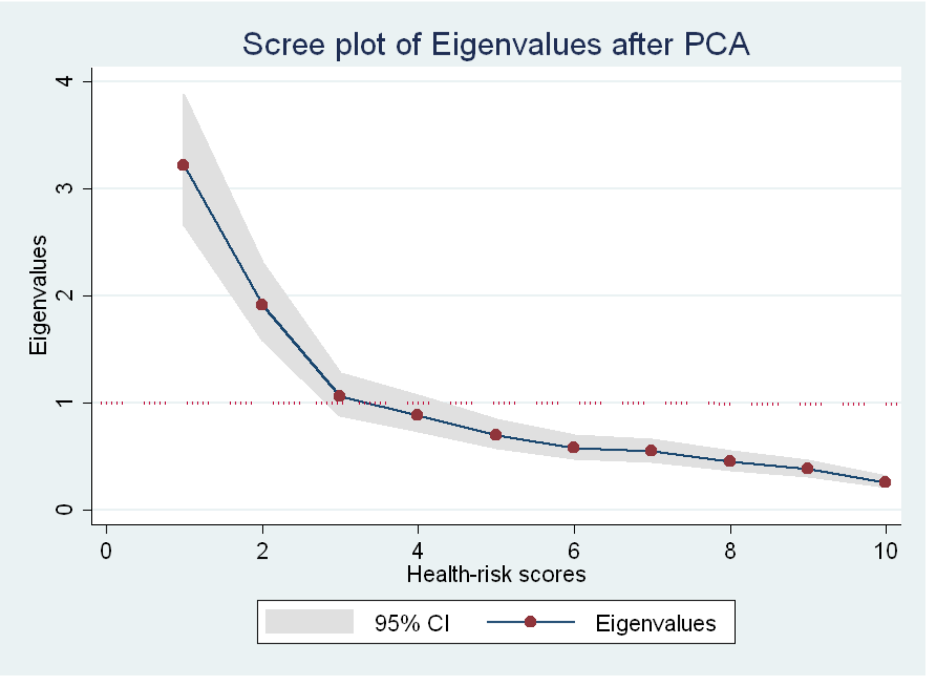

PCA, Principal Components Analysis; *Eigenvalue*, variance of the component; *95% CI*, Confidence interval.

Figure PAC 1. Scree Plot of the Eigenvalues.

Knowledge towards e-cigarettes and harm reduction among Public Health Residents in Europe

File 3

SUPPLEMENTARY TABLES

Table S1. Health risk scores for smoking products and components.

PRODUCT (*N*)	MEDIAN and RANGE
Tobacco cigarettes (n = 253)	10 (7 - 10)
Snus (n = 227)	9 (4 - 10)
Electronic cigarettes (n = 254)	7 (1 - 10)
NRT (n = 253)	4 (1 - 10)
Non-NRT oral medications (n = 247)	4 (1 - 10)
COMPONENT (*N*)	
Nicotine (n = 254)	7 (1 - 10)
Inhaled smoke (n = 254)	10 (3 - 10)
Carbon monoxide (n = 253)	10 (3 - 10)
Tar (n = 254)	9 (5 - 10)
Tobacco (n = 253)	9 (4 - 10)

NRT, nicotine replacement therapy; TAR, tobacco residue

Table S2. Differences in score for smoking products and components between smokers and non-smokers

	**Smokers (Current and Former) n = 57**	**Never Smokers n = 198**	
PRODUCT	MEDIAN and RANGE	MEDIAN and RANGE	*P*
Tobacco cigarettes	10 (8 - 10)	10 (7 - 10)	.83
Snus	8 (4 - 10)	9 (4 - 10)	.51
Electronic cigarettes	6 (2 - 10)	7 (1 - 10)	**.04**
NRT	3 (1 - 10)	4 (1 - 10)	.08
Non-NRT oral medications	4 (1 - 10)	4 (1 - 10)	.73
COMPONENT*			
Nicotine	7 (1 - 10)	7 (1 - 10)	.66
Inhaled smoke	10 (3 - 10)	10 (4 - 10)	.07
Carbon monoxide	10 (3 - 10)	10 (3 - 10)	.31
Tar (tobacco residue)	9 (5 - 10)	9 (5 - 10)	.96
Tobacco	9 (4 - 10)	9 (4 - 10)	.94

NRT, nicotine replacement therapy; TAR, tobacco residue

Table S3. Differences in score for smoking products and components for MPRH who attended specific training in smoking cessation during residency

	Specific training		
	Yes n = 51	No n = 203	*P*
PRODUCT	MEDIAN and RANGE	MEDIAN and RANGE	
Tobacco cigarettes	10 (8 - 10)	10 (7 - 10)	.67
Snus	9 (4 - 10)	8 (4 - 10)	.38
Electronic cigarettes	8 (1 - 10)	7 (2 - 10)	.31
NRT	3 (1 - 9)	4 (1 - 10)	**.01**
Non-NRT oral medications	3 (1 - 9)	4 (1 - 10)	.07
COMPONENT*			
Nicotine	8 (1 - 10)	7 (1 - 10)	.75
Inhaled smoke	10 (4 - 10)	10 (3 - 10)	.96
Carbon monoxide	10 (3 - 10)	10 (3 - 10)	.36
Tar (tobacco residue)	10 (7 - 10)	9 (5 - 10)	.17
Tobacco	9 (4 - 10)	8 (4 - 10)	**.03**

NRT, nicotine replacement therapy; TAR, tobacco residue

Table S4. Differences in risk scores for smoking products and components*

PRODUCT	**By country (n = 215§)** *P*	**By year of residency (n = 243)** *P*	**By setting of practice (n = 226§)** *P*

Tobacco cigarettes	.85	.51	.99
Snus	.23	**.05**	.66
Electronic cigarettes	**< .001**	.30	.55
NRT	**< .001**	.28	**.05**
Non-NRT oral medications	**.005**	.73	.10
COMPONENT			
Nicotine	**< .001**	.21	.39
Inhaled smoke	.18	.96	.34
Carbon monoxide	.12	.32	.70
Tar	.06	.31	**.02**
Tobacco	.12	.51	**.01**

* Studied by using Kruskal-Wallis H test
§ Observations categorized as "*others*" were excluded from the analysis
NRT, nicotine replacement therapy; TAR, tobacco residue

Table S5. Contribution of nicotine to diseases.

DISEASE *	N (%)
**Smoking-related diseases**	
Extremely important	72 (28.3)
Very important	75 (29.5)
Important	62 (24.4)
Unimportant	38 (15.0)
No contribution	7 (2.8)
**Lung cancer**	
Extremely important	55 (21.7)
Very important	44 (17.3)
Important	51 (20.1)
Unimportant	78 (30.7)
No contribution	26 (10.2)
**Cancer in other organs**	
Extremely important	31 (12.3)
Very important	65 (25.7)
Important	61 (24.1)
Unimportant	72 (28.5)
No contribution	24 (9.5)
**Atherosclerosis**	
Extremely important	46 (18.3)
Very important	75 (29.8)
Important	62 (24.6)
Unimportant	51 (20.2)
No contribution	18 (7.1)

** Number for each item may not add up to total number of study population due to missing value*

Table S6. Contribution of nicotine to diseases. Differences between smokers and non-smokers.

	Smokers (Current and Former) n = 57	Never Smokers n = 198	
DISEASE	N (%)	N (%)	*P*
**Smoking-related diseases**			.97
Extremely important	17 (29.8)	55 (27.9)	
Very important	18 (31.6)	57 (28.9)	
Important	14 (24.5)	48 (24.4)	
Unimportant	7 (12.3)	31 (15.7)	
No contribution	1 (1.8)	6 (3.1)	
**Lung cancer**			.12
Extremely important	10 (17.5)	45 (22.8)	
Very important	14 (24.5)	30 (15.2)	
Important	12 (21.1)	39 (19.8)	
Unimportant	12 (21.1)	66 (33.5)	

No contribution	9 (15.8)	17 (8.6)	
**Cancer in other organs**			.22
Extremely important	4 (7.0)	27 (13.8)	
Very important	20 (35.1)	45 (22.9)	
Important	25 (26.3)	46 (23.5)	
Unimportant	12 (21.1)	60 (20.6)	
No contribution	6 (10.5)	18 (8.2)	
**Atherosclerosis**			.36
Extremely important	9 (16.1)	37 (18.9)	
Very important	23 (41.1)	52 (26.5)	
Important	11 (19.6)	51 (26.0)	
Unimportant	9 (16.1)	42 (21.5)	
No contribution	4 (7.1)	14 (7.1)	

Table S7. Contribution of nicotine to diseases for MPRH who attended specific training in smoking cessation during residency.

	Specific Training		*P*
	Yes<br>n = 51	No<br>n = 203	
DISEASE	N (%)	N (%)	
**Smoking-related diseases**			
Extremely important	19 (37.3)	52 (25.7)	
Very important	11 (21.6)	64 (31.7)	
Important	9 (17.6)	53 (26.2)	.12
Unimportant	9 (17.6)	29 (14.4)	
No contribution	3 (5.9)	4 (2.0)	
**Lung cancer**			
Extremely important	11 (21.6)	43 (21.3)	
Very important	9 (17.6)	35 (17.3)	
Important	11 (21.6)	40 (19.8)	.86
Unimportant	17 (33.3)	61 (30.2)	
No contribution	3 (5.9)	23 (11.4)	
**Cancer in other organs**			
Extremely important	7 (13.7)	24 (11.9)	
Very important	13 (25.5)	51 (25.4)	
Important	14 (27.5)	47 (23.4)	.64
Unimportant	15 (29.4)	57 (28.4)	
No contribution	2 (3.9)	22 (10.9)	
**Atherosclerosis**			
Extremely important	8 (16.3)	38 (18.8)	
Very important	16 (32.7)	58 (28.7)	
Important	16 (32.7)	56 (22.8)	.42
Unimportant	6 (12.2)	45 (22.3)	
No contribution	3 (6.1)	15 (7.4)	

Table S8. Differences in contribution of nicotine to diseases.

	**By country (n = 215§)**	**By year of residency (n = 203)**	**By setting of practice (n = 226§)**
DISEASE	*P*	*P*	*P*
Smoking-related diseases	.18	**.003**	**.01**
Lung cancer	.60	.99	.38
Cancer in other organs	.56	.92	.68
Atherosclerosis	.32	.97	.46

§ Observations categorized as "*others*" were excluded from the analysis

Knowledge towards e-cigarettes and harm reduction among Public Health Residents in Europe

Table S9. Participants' responses on e-cigarettes and tobacco harm reduction. Differences between smokers and non-smokers.

QUESTIONS *	Smokers (Current and Former) n = 57	Never Smokers n = 198	*P*
The health risk of nicotine replacement therapies compared to smoking is:			.34
Higher	1 (1.7)	3 (1.5)	
Equal	6 (10.5)	10 (5.1)	
Lower	47 (82.5)	176 (89.8)	
Do not know	3 (5.3)	7 (3.6)	
The health risk of electronic cigarettes compared to smoking is:			.75
Higher	1 (1.8)	9 (4.6)	
Equal	19 (33.9)	55 (28.1)	
Lower	32 (57.2)	117 (59.7)	
Do not know	4 (7.1)	15 (7.6)	
The harmful effect of electronic cigarettes is due to the diethylene glycol:			.36
Yes			
No	13 (23.2)	44 (22.3)	
Do not know	9 (16.1)	19 (9.4)	
	34 (60.7)	134 (68.0)	
Electronic cigarettes can generate addiction:			.89
Yes	49 (87.5)	165 (84.2)	
No	2 (3.6)	8 (4.1)	
Do not know	5 (8.9)	23 (11.7)	
The dependence potential of electronic cigarettes compared to smoking is:			.81
Higher	5 (8.9)	21 (10.6)	
Equal	28 (50.0)	102 (51.8)	
Lower	17 (30.4)	48 (24.4)	
Do not know	6 (10.7)	26 (13.2)	
Electronic cigarettes are more expensive than normal tobacco:			.07
Yes	16 (28.6)	67 (34.0)	
No	25 (44.6)	56 (28.4)	
Do not know	15 (26.8)	74 (37.6)	
Electronic cigarettes are safer than tobacco:			.29
Yes	21 (37.5)	90 (45.9)	
No	21 (37.5)	74 (37.7)	
Do not know	14 (25.0)	32 (16.3)	
Electronic cigarettes are effective devices for smoking cessation:			.11
Yes			
No	23 (41.8)	57 (28.9)	
Do not know	22 (40.0)	109 (55.3)	
	10 (18.2)	31 (15.7)	
As a Public Health professional, would you recommend the electronic cigarette as smoking cessation aid to a patient?			.47
Yes	19 (33.9)	50 (25.5)	
No	33 (58.9)	128 (65.3)	
Do not know	4 (7.2)	18 (9.2)	
As a Public Health professional, would you recommend the electronic cigarette to a patient for reducing the number of smoked cigarettes?			.30
Yes			
No	26 (46.4)	84 (42.7)	
Do not know	22 (39.3)	96 (48.7)	
	8 (14.3)	17 (8.6)	
Do you think that the concomitant use of electronic cigarettes and tobacco will effectively reduce the number of smoked cigarettes?			.21
Yes			

No	15 (26.8)	77 (39.1)	
Do not know	28 (50.0)	87 (44.2)	
	13 (23.2)	33 (16.7)	
Do you think that medical community and healthcare workers should take a position in favour of the electronic cigarettes?			.83
Yes	18 (32.1)	55 (28.1)	
No	28 (20.0)	102 (52.0)	
Do not know	10 (17.8)	39 (19.9)	
Do you think that electronic cigarettes should be prohibited?			.98
Yes	14 (25.0)	52 (26.4)	
No	32 (57.1)	111 (56.3)	
Do not know	10 (17.9)	34 (17.3)	
Have you heard of modified-risk tobacco?			.92
Yes	14 (25.0)	48 (24.4)	
No	42 (75.0)	149 (75.6)	
The health risk of modified-risk tobacco products compared to smoking is:	2 (3.6)	4 (2.0)	.41
Higher	8 (14.5)	21 (10.8)	
Equal	4 (7.3)	29 (15.0)	
Lower	41 (74.6)	140 (72.2)	
Do not know			
The health risk of modified-risk tobacco products compared to electronic cigarettes is:			.51
Higher	4 (7.3)	19 (9.7)	
Equal	6 (10.9)	19 (9.7)	
Lower	0 (0.0)	8 (4.2)	
Do not know	45 (81.9)	149 (76.4)	
As a Public Health professional, would you recommend modified-risk tobacco products to reduce tobacco-related problems?			.83
Yes			
No	3 (5.4)	11 (5.6)	
Do not know	11 (20.0)	48 (24.6)	
	41 (74.6)	136 (69.8)	

** Number for each item may not add up to total number of study population due to missing value*

Table S10. Participants' responses on e-cigarettes and tobacco harm reduction. Differences for MPRH who attended specific training in smoking cessation during residency.

	Specific training		
	Yes<br>n = 51	No<br>n = 203	*P*
The health risk of nicotine replacement therapies compared to smoking is:			.66
Higher	1 (2.0)	3 (1.5)	
Equal	3 (6.0)	13 (6.4)	
Lower	43 (86.0)	180 (88.7)	
Do not know	3 (6.0)	7 (3.4)	
The health risk of electronic cigarettes compared to smoking is:			.79
Higher	1 (2.0)	9 (4.5)	
Equal	17 (33.3)	57 (28.4)	
Lower	29 (56.9)	120 (59.7)	
Do not know	4 (7.8)	15 (7.5)	
The harmful effect of electronic cigarettes is due to the diethylene glycol:			.56
Yes			
No	9 (17.6)	48 (23.9)	
Do not know	5 (9.8)	23 (11.4)	
	37 (72.6)	130 (64.7)	
Electronic cigarettes can generate addiction:			.73
Yes	42 (82.4)	178 (86.0)	
No	2 (3.9)	8 (4.0)	
Do not know	7 (13.7)	20 (10.0)	

The dependence potential of electronic cigarettes compared to smoking is:			.30
Higher	2 (2.9)	24 (11.9)	
Equal	31 (60.8)	99 (49.3)	
Lower	12 (23.5)	52 (25.9)	
Do not know	6 (11.8)	26 (12.9)	
Electronic cigarettes are more expensive than normal tobacco:			.37
Yes	21 (41.2)	62 (30.9)	
No	14 (27.4)	67 (33.3)	
Do not know	16 (31.4)	72 (35.8)	
Electronic cigarettes are safer than tobacco:			.14
Yes	22 (43.1)	88 (44.0)	
No	24 (47.1)	71 (35.5)	
Do not know	5 (9.8)	41 (20.5)	
Electronic cigarettes are effective devices for smoking cessation:			.12
Yes			
No	13 (25.5)	66 (33.0)	
Do not know	33 (64.7)	98 (49.0)	
	5 (9.8)	36 (18.0)	
As a Public Health professional, would you recommend the electronic cigarette as smoking cessation aid to a patient?			**.04**
Yes	8 (16.0)	60 (44.8)	
No	34 (68.0)	127 (63.2)	
Do not know	8 (16.0)	14 (29.8)	
As a Public Health professional, would you recommend the electronic cigarette to a patient for reducing the number of smoked cigarettes?			.47
Yes	19 (37.3)	90 (44.8)	
No	25 (49.0)	93 (46.3)	
Do not know	7 (13.7)	18 (8.9)	
Do you think that the concomitant use of electronic cigarettes and tobacco will effectively reduce the number of smoked cigarettes?			.82
Yes			
No	18 (35.3)	73 (36.3)	
Do not know	25 (49.0)	90 (44.8)	
	8 (15.7)	38 (18.9)	
Do you think that medical community and healthcare workers should take a position in favour of the electronic cigarettes?			.48
Yes	14 (27.4)	58 (29.0)	
No	24 (47.1)	106 (53.0)	
Do not know	13 (25.5)	36 (18.0)	
Do you think that electronic cigarettes should be prohibited?			.99
Yes	13 (25.5)	53 (26.4)	
No	29 (56.9)	113 (56.2)	
Do not know	9 (17.6)	35 (17.4)	
Have you heard of modified-risk tobacco?			.60
Yes	14 (27.5)	48 (23.9)	
No	37 (72.5)	153 (76.1)	
The health risk of modified-risk tobacco products compared to smoking is:			.85
Higher	0 (0.0)	6 (3.0)	
Equal	6 (12.0)	23 (11.6)	
Lower	7 (14.0)	26 (13.1)	
Do not know	37 (74.0)	143 (72.2)	
The health risk of modified-risk tobacco products compared to electronic cigarettes is:			.10
Higher	9 (17.7)	14 (7.1)	
Equal	3 (5.9)	22 (11.1)	
Lower	2 (3.9)	6 (3.0)	
Do not know	37 (72.5)	156 (78.8)	
As a Public Health professional, would you recommend modified-risk tobacco products to reduce tobacco-related problems?			.76

Yes		
No	2 (3.9)	12 (6.1)
Do not know	14 (27.4)	45 (22.7)
	35 (68.6)	141 (71.2)

** Number for each item may not add up to total number of study population due to missing value*

Table S11. Participants' responses on e-cigarettes and tobacco harm reduction. Differences in responses amongst MRPH by...

QUESTIONS *	By country (n = 215[§])	By year of residency (n = 203)	By setting of practice (n = 226[§])
The health risk of nicotine replacement therapies compared to smoking is:	.009	.44	.17
Higher			
Equal			
Lower			
Do not know			
The health risk of electronic cigarettes compared to smoking is:	.86	.77	.92
Higher			
Equal			
Lower			
Do not know			
The harmful effect of electronic cigarettes is due to the diethylene glycol:	.15	.88	.91
Yes			
No			
Do not know			
Electronic cigarettes can generate addiction:	.26	.71	.12
Yes			
No			
Do not know			
The dependence potential of electronic cigarettes compared to smoking is:	.83	.71	.95
Higher			
Equal			
Lower			
Do not know			
Electronic cigarettes are more expensive than normal tobacco:	.28	.52	.57
Yes			
No			
Do not know			
Electronic cigarettes are safer than tobacco:	.22	.60	.16
Yes			
No			
Do not know			
Electronic cigarettes are effective devices for smoking cessation:	.40	.15	.09
Yes			
No			
Do not know			
As a Public Health professional, would you recommend the electronic cigarette as smoking cessation aid to a patient?	.64	.75	.52
Yes			
No			
Do not know			
As a Public Health professional, would you recommend the electronic cigarette to a patient for reducing the number of smoked cigarettes?	.70	.93	.63
Yes			
No			
Do not know			
Do you think that the concomitant use of electronic cigarettes and tobacco will effectively reduce the number of smoked cigarettes?	.98	.27	.64
Yes			
No			

Do not know			
Do you think that medical community and healthcare workers should take a position in favour of the electronic cigarettes?	.13	.92	.63
Yes			
No			
Do not know			
Do you think that electronic cigarettes should be prohibited?	.71	.98	.69
Yes			
No			
Do not know			
Have you heard of modified-risk tobacco?	.07	.74	.97
Yes			
No			
The health risk of modified-risk tobacco products compared to smoking is:	.09	.79	.74
Higher			
Equal			
Lower			
Do not know			
The health risk of modified-risk tobacco products compared to electronic cigarettes is:	.09	.84	.40
Higher			
Equal			
Lower			
Do not know			
As a Public Health professional, would you recommend modified-risk tobacco products to reduce tobacco-related problems?	.36	.54	.97
Yes			
No			
Do not know			

* Number for each item may not add up to total number of study population due to missing value
§ Observations categorized as "*others*" were excluded from the analysis
