# Supplementary material for: Knowledge about E-Cigarettes and Tobacco Harm Reduction among Public Health Residents in Europe
Source: Int J Environ Res Public Health. 2019 Jun 12;16(12):2071. doi: 10.3390/ijerph16122071 (PMC6617304; doi:10.3390/ijerph16122071)
Supplement: Supplementary file 1 [file ijerph-16-02071-s001.zip › ijerph-510448-supplementary/ijerph-510448-supplementary.doc]

File 1

Survey

A1. *Participant characteristics*

1. **Gender:** □ M □ F

2. **Year of birth:** *in years*

3. **Country of residency:** [Bosnia / Croatia / France / Ireland / Italy / Malta / Moldova / The Netherlands / Poland / Portugal / Slovenia / Spain / United Kingdom / Other, *specify*:…

4. **Residency year:** □ 1 □ 2 □ 3 □ 4 □ 5 □ 6

5. **Setting of practice:** □ University □ Hospital □ Other facilities: *indicate*…

6. **Have you attended specific training in smoking cessation during residency**:

□ Yes □ No

A2. *Smoking status and habit*

7. **Current smoker:** □ Yes	□ No	□ Former smoker

7.a - ***If current smoker, which of the following tobacco products do you use?***

□ Cigarettes □ Electronic Cigarettes □ Other*: *indicate*…

*(including pipe tobacco, waterpipe tobacco, roll-your-own tobacco, cigars, chewing tobacco, nasal tobacco, tobacco for oral use, or other than the listed tobacco related products)

7.b - ***If cigarettes smoker:*** *indicate the number per day*

7.c - ***If current smoker, are* you considering quitting:** □ Yes □ No

B. *Knowledge and attitudes*

***Using the scale below from 1 (low risk) to 10 (high risk), please indicate the risk score for health of the following products and smoking components***

**8. Health risk score for products:**

| Tobacco cigarettes: | (low risk) □ 1 □ 2 □ 3 □ 4 □ 5 □ 6 □ 7 □ 8 □ 9 □ 10 (high risk) |
| --- | --- |
| Snus: | (low risk) □ 1 □ 2 □ 3 □ 4 □ 5 □ 6 □ 7 □ 8 □ 9 □ 10 (high risk) |
| Electronic cigarettes: | (low risk) □ 1 □ 2 □ 3 □ 4 □ 5 □ 6 □ 7 □ 8 □ 9 □ 10 (high risk) |
| Nicotine replacement therapy (NRT): | (low risk) □ 1 □ 2 □ 3 □ 4 □ 5 □ 6 □ 7 □ 8 □ 9 □ 10 (high risk) |
| Non-NRT oral medications: | (low risk) □ 1 □ 2 □ 3 □ 4 □ 5 □ 6 □ 7 □ 8 □ 9 □ 10 (high risk) |

9. **Health risk score for smoking components:**

| Nicotine: | (low risk) □ 1 □ 2 □ 3 □ 4 □ 5 □ 6 □ 7 □ 8 □ 9 □ 10 (high risk) |
| --- | --- |
| Inhaled smoke: | (low risk) □ 1 □ 2 □ 3 □ 4 □ 5 □ 6 □ 7 □ 8 □ 9 □ 10 (high risk) |
| Carbon monoxide: | (low risk) □ 1 □ 2 □ 3 □ 4 □ 5 □ 6 □ 7 □ 8 □ 9 □ 10 (high risk) |
| Tar: | (low risk) □ 1 □ 2 □ 3 □ 4 □ 5 □ 6 □ 7 □ 8 □ 9 □ 10 (high risk) |
| Tobacco: | (low risk) □ 1 □ 2 □ 3 □ 4 □ 5 □ 6 □ 7 □ 8 □ 9 □ 10 (high risk) |

***Using the scale below, please indicate how important you believe each of the following statements to be***

10. **Contribution of nicotine to smoking-related diseases:**

□ Extremely important	□ Very important	□ Important

□ Unimportant	□ No contribution

11. **Contribution of nicotine to lung cancer:**

□ Extremely important	□ Very important	□ Important

□ Unimportant	□ No contribution

12. **Contribution of nicotine to cancer in other organs:**

□ Extremely important	□ Very important	□ Important

□ Unimportant	□ No contribution

13. **Contribution of nicotine to atherosclerosis:**

□ Extremely important	□ Very important	□ Important

□ Unimportant	□ No contribution

***You will now be asked about your thoughts and knowledge on a series of statements***

14. **The health risk of nicotine replacement therapies compared to smoking is:**

□ Higher □ Equal □ Lower □ Do not know

15. **The health risk of electronic cigarettes compared to smoking is:**

□ Higher □ Equal □ Lower □ Do not know

16. **The harmful effect of electronic cigarettes is due to the diethylene glycol:**

□ Yes □ No □ Do not know

17. **Electronic cigarettes can generate addiction:**

□ Yes □ No □ Do not know

18. **The dependence potential of electronic cigarettes compared to smoking is:**

□ Higher □ Equal □ Lower □ Do not know

19. **Electronic cigarettes are more expensive than normal tobacco:**

□ Yes □ No □ Do not know

20. **Electronic cigarettes are safer than tobacco:**

□ Yes □ No □ Do not know

21. **Electronic cigarettes are effective devices for smoking cessation:**

□ Yes □ No □ Do not know

22. **As a Public Health professional, would you recommend the electronic cigarette as smoking cessation aid to a patient?**

□ Yes □ No □ Do not know

23. **As a Public Health professional, would you recommend the electronic cigarette to a patient for reducing the number of smoked cigarettes?**

□ Yes □ No □ Do not know

24. **Do you think that the concomitant use of electronic cigarettes and tobacco will effectively reduce the number of smoked cigarettes?**

□ Yes □ No □ Do not know

25. **Do you think that medical community and healthcare workers should take a position in favour of the electronic cigarettes?**

□ Yes □ No □ Do not know

26. **Do you think that electronic cigarettes should be prohibited?**

□ Yes □ No □ Do not know

27. **Have you heard of modified-risk tobacco?**

□ Yes □ No

28. **The health risk of modified-risk tobacco products compared to smoking is:**

□ Higher □ Equal □ Lower □ Do not know

29. **The health risk of modified-risk tobacco products compared to electronic cigarettes is:**

□ Higher □ Equal □ Lower □ Do not know

30. **As a Public Health professional, would you recommend modified-risk tobacco products to reduce tobacco-related problems?**

□ Yes □ No □ Do not know

File 2

Results of Principal Components Analysis

In order to check content validity of the survey instrument designed for the purposes of the study, Principal Components Analysis (PCA) were conducted on variables that point back to the health risk scores for smoking products and components:

1. *Tobacco cigarettes*
2. *Electronic cigarettes*
3. *Snus*
4. *Nicotine replacement therapy (NRT)*
5. *Non-NRT oral medications*
6. *Nicotine*
7. *Inhaled smoke*
8. *Carbon monoxide*
9. *Tobacco*
10. *Tobacco residue*

Results are presented in Table PCA 1 and Figure PCA 1.

**Table PCA 1.** Results of Principal Components Analysis and Factor Analysis.

| ***Eigenvalues of the principal components*** | | | | | | | | | | | |
| --- | --- | --- | --- | --- | --- | --- | --- | --- | --- | --- | --- |
|  | **Eigenvalue** | | | **Difference** | | **Proportion of variance explained** | | | **Cumulative variance explained** | | |
| 1 | 3.22 | | | 1.31 | | 0.32 | | | 0.32 | | |
| 2 | 1.91 | | | 0.85 | | 0.19 | | | 0.51 | | |
| 3 | 1.06 | | | 0.17 | | 0.11 | | | 0.62 | | |
| 4 | 0.89 | | | 0.19 | | 0.09 | | | 0.71 | | |
| 5 | 0.70 | | | 0.12 | | 0.07 | | | 0.78 | | |
| 6 | 0.58 | | | 0.03 | | 0.06 | | | 0.84 | | |
| 7 | 0.55 | | | 0.09 | | 0.05 | | | 0.89 | | |
| 8 | 0.46 | | | 0.08 | | 0.05 | | | 0.94 | | |
| 9 | 0.38 | | | 0.12 | | 0.03 | | | 0.97 | | |
| 10 | 0.26 | | | - | | 0.03 | | | 1.00 | | |
| ***Factor loadings*** | | | | | | | | |  | | |
| Variables | | Factor1 | | | Factor2 | | | Factor3 | | |  |
| 1 | | | 0.30 | | 0.31 | | - 0.19 | | |  | |
| 2 | | | 0.39 | | - 0.12 | | - 0.20 | | |  | |
| 3 | | | 0.39 | | - 0.07 | | - 0.16 | | |  | |
| 4 | | | 0.30 | | - 0.48 | | 0.29 | | |  | |
| 5 | | | 0.26 | | - 0.44 | | 0.43 | | |  | |
| 6 | | | 0.35 | | - 0.25 | | - 0.21 | | |  | |
| 7 | | | 0.31 | | 0.27 | | - 0.09 | | |  | |
| 8 | | | 0.34 | | 0.18 | | - 0.29 | | |  | |
| 9 | | | 0.28 | | 0.36 | | 0.35 | | |  | |
| 10 | | | 0.18 | | 0.41 | | 0.60 | | |  | |
| *Eigenvalue* represents the variance of the component and is considerate to be critical for values > 1. All the values add to the sum of the number of the variables. The *Difference* is the size of a component’s eigenvalue and the next component’s eigenvalue. Thus, the first three principal components with eigenvalues > 1 explained the 67% of the information in the data set.  Factor loadings reflected the multiplicity of the survey. | | | | | | | | | | | |

*PCA*, Principal Components Analysis; *Eigenvalue*, variance of the component; *95% CI*, Confidence interval.

**Figure PAC 1.** Scree Plot of the Eigenvalues.

File 3

Supplementary Tables

**Table S1**. Health risk scores for smoking products and components.

| **Product (*N*)** | **Median and Range** |
| --- | --- |
| Tobacco cigarettes (n = 253) | 10 (7 - 10) |
| Snus (n = 227) | 9 (4 - 10) |
| Electronic cigarettes (n = 254) | 7 (1 - 10) |
| NRT (n = 253) | 4 (1 - 10) |
| Non-NRT oral medications (n = 247) | 4 (1 - 10) |
| Component (*N*) |  |
| Nicotine (n = 254) | 7 (1 - 10) |
| Inhaled smoke (n = 254) | 10 (3 - 10) |
| Carbon monoxide (n = 253) | 10 (3 - 10) |
| Tar (n = 254) | 9 (5 - 10) |
| Tobacco (n = 253) | 9 (4 - 10) |
| *NRT, nicotine replacement therapy; Tar, tobacco residue* | |

**Table S2.** Differences in score for smoking products and components between smokers and non-smokers

|  | **Smokers (Current and Former)**  **n = 57** | **Never Smokers**  **n = 198** |  |
| --- | --- | --- | --- |
| Product | Median and Range | Median and Range | *P* |
| Tobacco cigarettes | 10 (8 - 10) | 10 (7 - 10) | .83 |
| Snus | 8 (4 - 10) | 9 (4 - 10) | .51 |
| Electronic cigarettes | 6 (2 - 10) | 7 (1 - 10) | **.04** |
| NRT | 3 (1 - 10) | 4 (1 - 10) | .08 |
| Non-NRT oral medications | 4 (1 - 10) | 4 (1 - 10) | .73 |
| Component* |  |  |  |
| Nicotine | 7 (1 - 10) | 7 (1 - 10) | .66 |
| Inhaled smoke | 10 (3 - 10) | 10 (4 - 10) | .07 |
| Carbon monoxide | 10 (3 - 10) | 10 (3 - 10) | .31 |
| Tar (tobacco residue) | 9 (5 - 10) | 9 (5 - 10) | .96 |
| Tobacco | 9 (4 - 10) | 9 (4 - 10) | .94 |
| *NRT, nicotine replacement therapy; Tar, tobacco residue* | | |  |

**Table S3.** Differences in score for smoking products and components for MPRH who attended specific training in smoking cessation during residency

|  | ***Specific training*** | |  |
| --- | --- | --- | --- |
|  | Yes  n = 51 | No  n = 203 | *P* |
| Product | Median and Range | Median and Range |  |
| Tobacco cigarettes | 10 (8 - 10) | 10 (7 - 10) | .67 |
| Snus | 9 (4 - 10) | 8 (4 - 10) | .38 |
| Electronic cigarettes | 8 (1 - 10) | 7 (2 - 10) | .31 |
| NRT | 3 (1 - 9) | 4 (1 - 10) | **.01** |
| Non-NRT oral medications | 3 (1 - 9) | 4 (1 - 10) | .07 |
| Component* |  |  |  |
| Nicotine | 8 (1 - 10) | 7 (1 - 10) | .75 |
| Inhaled smoke | 10 (4 - 10) | 10 (3 - 10) | .96 |
| Carbon monoxide | 10 (3 - 10) | 10 (3 - 10) | .36 |
| Tar (tobacco residue) | 10 (7 - 10) | 9 (5 - 10) | .17 |
| Tobacco | 9 (4 - 10) | 8 (4 - 10) | **.03** |
| *NRT, nicotine replacement therapy; Tar, tobacco residue* | | |  |

**Table S4.** Differences in risk scores for smoking products and components*

| **Product** | **By country**  **(n = 215*^§^*)**  ***P*** | **By year of residency**  **(n = 243)**  ***P*** | **By setting of practice**  **(n = 226*^§^*)**  ***P*** |
| --- | --- | --- | --- |
| Tobacco cigarettes | .85 | .51 | .99 |
| Snus | .23 | **.05** | .66 |
| Electronic cigarettes | **< .001** | .30 | .55 |
| NRT | **< .001** | .28 | **.05** |
| Non-NRT oral medications | **.005** | .73 | .10 |
| Component |  |  |  |
| Nicotine | **< .001** | .21 | .39 |
| Inhaled smoke | .18 | .96 | .34 |
| Carbon monoxide | .12 | .32 | .70 |
| Tar | .06 | .31 | **.02** |
| Tobacco | .12 | .51 | **.01** |
| *** Studied by using Kruskal-Wallis H test  *^§^* Observations categorized as “*others*” were excluded from the analysis  *NRT, nicotine replacement therapy; Tar, tobacco residue* | | | |

**Table S5.** Contribution of nicotine to diseases.

| Disease * | n (%) |
| --- | --- |
| **Smoking-related diseases**  *Extremely important*  *Very important*  *Important*  *Unimportant*  *No contribution* | 72 (28.3)  75 (29.5)  62 (24.4)  38 (15.0)  7 (2.8) |
| **Lung cancer**  *Extremely important*  *Very important*  *Important*  *Unimportant*  *No contribution* | 55 (21.7)  44 (17.3)  51 (20.1)  78 (30.7)  26 (10.2) |
| **Cancer in other organs**  *Extremely important*  *Very important*  *Important*  *Unimportant*  *No contribution* | 31 (12.3)  65 (25.7)  61 (24.1)  72 (28.5)  24 (9.5) |
| **Atherosclerosis**  *Extremely important*  *Very important*  *Important*  *Unimportant*  *No contribution* | 46 (18.3)  75 (29.8)  62 (24.6)  51 (20.2)  18 (7.1) |
| ** Number for each item may not add up to total number of study population due to missing value* | |

**Table S6. Contribution of nicotine to diseases. Differences between smokers and non-smokers.**

|  | Smokers (Current and Former)  n = 57 | Never Smokers  n = 198 |  |
| --- | --- | --- | --- |
| Disease | n (%) | n (%) | *p* |
| **Smoking-related diseases**  *Extremely important*  *Very important*  *Important*  *Unimportant*  *No contribution* | 17 (29.8)  18 (31.6)  14 (24.5)  7 (12.3)  1 (1.8) | 55 (27.9)  57 (28.9)  48 (24.4)  31 (15.7)  6 (3.1) | .97 |
| **Lung cancer**  *Extremely important*  *Very important*  *Important*  *Unimportant*  *No contribution* | 10 (17.5)  14 (24.5)  12 (21.1)  12 (21.1)  9 (15.8) | 45 (22.8)  30 (15.2)  39 (19.8)  66 (33.5)  17 (8.6) | .12 |
| **Cancer in other organs**  *Extremely important*  *Very important*  *Important*  *Unimportant*  *No contribution* | 4 (7.0)  20 (35.1)  25 (26.3)  12 (21.1)  6 (10.5) | 27 (13.8)  45 (22.9)  46 (23.5)  60 (20.6)  18 (8.2) | .22 |
| **Atherosclerosis**  *Extremely important*  *Very important*  *Important*  *Unimportant*  *No contribution* | 9 (16.1)  23 (41.1)  11 (19.6)  9 (16.1)  4 (7.1) | 37 (18.9)  52 (26.5)  51 (26.0)  42 (21.5)  14 (7.1) | .36 |

**Table S7.** Contribution of nicotine to diseases for MPRH who attended specific training in smoking cessation during residency.

|  | *Specific Training* | |  |
| --- | --- | --- | --- |
|  | Yes  n = 51 | No  n = 203 | *p* |
| Disease | n (%) | n (%) |  |
| **Smoking-related diseases**  *Extremely important*  *Very important*  *Important*  *Unimportant*  *No contribution* | 19 (37.3)  11 (21.6)  9 (17.6)  9 (17.6)  3 (5.9) | 52 (25.7)  64 (31.7)  53 (26.2)  29 (14.4)  4 (2.0) | .12 |
| **Lung cancer**  *Extremely important*  *Very important*  *Important*  *Unimportant*  *No contribution* | 11 (21.6)  9 (17.6)  11 (21.6)  17 (33.3)  3 (5.9) | 43 (21.3)  35 (17.3)  40 (19.8)  61 (30.2)  23 (11.4) | .86 |
| **Cancer in other organs**  *Extremely important*  *Very important*  *Important*  *Unimportant*  *No contribution* | 7 (13.7)  13 (25.5)  14 (27.5)  15 (29.4)  2 (3.9) | 24 (11.9)  51 (25.4)  47 (23.4)  57 (28.4)  22 (10.9) | .64 |
| **Atherosclerosis**  *Extremely important*  *Very important*  *Important*  *Unimportant*  *No contribution* | 8 (16.3)  16 (32.7)  16 (32.7)  6 (12.2)  3 (6.1) | 38 (18.8)  58 (28.7)  56 (22.8)  45 (22.3)  15 (7.4) | .42 |

**Table S8.** Differences in contribution of nicotine to diseases.

|  | **By country**  **(n = 215*^§^*)** | **By year of residency**  **(n = 203)** | **By setting of practice**  **(n = 226*^§^*)** |
| --- | --- | --- | --- |
| Disease | *p* | *p* | *p* |
| Smoking-related diseases | .18 | **.003** | **.01** |
| Lung cancer | .60 | .99 | .38 |
| Cancer in other organs | .56 | .92 | .68 |
| Atherosclerosis | .32 | .97 | .46 |
| *^§^* Observations categorized as “*others*” were excluded from the analysis | | | |

**Table S9.** Participants’ responses on e-cigarettes and tobacco harm reduction. Differences between smokers and non-smokers.

| **Questions *** | **Smokers (Current and Former)**  **n = 57** | **Never Smokers**  **n = 198** | ***P*** |
| --- | --- | --- | --- |
| The health risk of nicotine replacement therapies compared to smoking is:  *Higher*  *Equal*  *Lower*  *Do not know* | 1 (1.7)  6 (10.5)  47 (82.5)  3 (5.3) | 3 (1.5)  10 (5.1)  176 (89.8)  7 (3.6) | .34 |
| The health risk of electronic cigarettes compared to smoking is:  *Higher*  *Equal*  *Lower*  *Do not know* | 1 (1.8)  19 (33.9)  32 (57.2)  4 (7.1) | 9 (4.6)  55 (28.1)  117 (59.7)  15 (7.6) | .75 |
| The harmful effect of electronic cigarettes is due to the diethylene glycol:  *Yes*  *No*  *Do not know* | 13 (23.2)  9 (16.1)  34 (60.7) | 44 (22.3)  19 (9.4)  134 (68.0) | .36 |
| Electronic cigarettes can generate addiction:  *Yes*  *No*  *Do not know* | 49 (87.5)  2 (3.6)  5 (8.9) | 165 (84.2)  8 (4.1)  23 (11.7) | .89 |
| The dependence potential of electronic cigarettes compared to smoking is:  *Higher*  *Equal*  *Lower*  *Do not know* | 5 (8.9)  28 (50.0)  17 (30.4)  6 (10.7) | 21 (10.6)  102 (51.8)  48 (24.4)  26 (13.2) | .81 |
| Electronic cigarettes are more expensive than normal tobacco:  *Yes*  *No*  *Do not know* | 16 (28.6)  25 (44.6)  15 (26.8) | 67 (34.0)  56 (28.4)  74 (37.6) | .07 |
| Electronic cigarettes are safer than tobacco:  *Yes*  *No*  *Do not know* | 21 (37.5)  21 (37.5)  14 (25.0) | 90 (45.9)  74 (37.7)  32 (16.3) | .29 |
| Electronic cigarettes are effective devices for smoking cessation:  *Yes*  *No*  *Do not know* | 23 (41.8)  22 (40.0)  10 (18.2) | 57 (28.9)  109 (55.3)  31 (15.7) | .11 |
| As a Public Health professional, would you recommend the electronic cigarette as smoking cessation aid to a patient?  *Yes*  *No*  *Do not know* | 19 (33.9)  33 (58.9)  4 (7.2) | 50 (25.5)  128 (65.3)  18 (9.2) | .47 |
| As a Public Health professional, would you recommend the electronic cigarette to a patient for reducing the number of smoked cigarettes?  *Yes*  *No*  *Do not know* | 26 (46.4)  22 (39.3)  8 (14.3) | 84 (42.7)  96 (48.7)  17 (8.6) | .30 |
| Do you think that the concomitant use of electronic cigarettes and tobacco will effectively reduce the number of smoked cigarettes?  *Yes*  *No*  *Do not know* | 15 (26.8)  28 (50.0)  13 (23.2) | 77 (39.1)  87 (44.2)  33 (16.7) | .21 |
| Do you think that medical community and healthcare workers should take a position in favour of the electronic cigarettes?  *Yes*  *No*  *Do not know* | 18 (32.1)  28 (20.0)  10 (17.8) | 55 (28.1)  102 (52.0)  39 (19.9) | .83 |
| Do you think that electronic cigarettes should be prohibited?  *Yes*  *No*  *Do not know* | 14 (25.0)  32 (57.1)  10 (17.9) | 52 (26.4)  111 (56.3)  34 (17.3) | .98 |
| Have you heard of modified-risk tobacco?  *Yes*  *No* | 14 (25.0)  42 (75.0) | 48 (24.4)  149 (75.6) | .92 |
| The health risk of modified-risk tobacco products compared to smoking is:  *Higher*  *Equal*  *Lower*  *Do not know* | 2 (3.6)  8 (14.5)  4 (7.3)  41 (74.6) | 4 (2.0)  21 (10.8)  29 (15.0)  140 (72.2) | .41 |
| The health risk of modified-risk tobacco products compared to electronic cigarettes is:  *Higher*  *Equal*  *Lower*  *Do not know* | 4 (7.3)  6 (10.9)  0 (0.0)  45 (81.9) | 19 (9.7)  19 (9.7)  8 (4.2)  149 (76.4) | .51 |
| As a Public Health professional, would you recommend modified-risk tobacco products to reduce tobacco-related problems?  *Yes*  *No*  *Do not know* | 3 (5.4)  11 (20.0)  41 (74.6) | 11 (5.6)  48 (24.6)  136 (69.8) | .83 |
| ** Number for each item may not add up to total number of study population due to missing value* | | | |

**Table S10.** Participants’ responses on e-cigarettes and tobacco harm reduction. Differences for MPRH who attended specific training in smoking cessation during residency.

|  | *Specific training* | |  |
| --- | --- | --- | --- |
|  | Yes  n = 51 | No  n = 203 | *P* |
| The health risk of nicotine replacement therapies compared to smoking is:  *Higher*  *Equal*  *Lower*  *Do not know* | 1 (2.0)  3 (6.0)  43 (86.0)  3 (6.0) | 3 (1.5)  13 (6.4)  180 (88.7)  7 (3.4) | .66 |
| The health risk of electronic cigarettes compared to smoking is:  *Higher*  *Equal*  *Lower*  *Do not know* | 1 (2.0)  17 (33.3)  29 (56.9)  4 (7.8) | 9 (4.5)  57 (28.4)  120 (59.7)  15 (7.5) | .79 |
| The harmful effect of electronic cigarettes is due to the diethylene glycol:  *Yes*  *No*  *Do not know* | 9 (17.6)  5 (9.8)  37 (72.6) | 48 (23.9)  23 (11.4)  130 (64.7) | .56 |
| Electronic cigarettes can generate addiction:  *Yes*  *No*  *Do not know* | 42 (82.4)  2 (3.9)  7 (13.7) | 178 (86.0)  8 (4.0)  20 (10.0) | .73 |
| The dependence potential of electronic cigarettes compared to smoking is:  *Higher*  *Equal*  *Lower*  *Do not know* | 2 (2.9)  31 (60.8)  12 (23.5)  6 (11.8) | 24 (11.9)  99 (49.3)  52 (25.9)  26 (12.9) | .30 |
| Electronic cigarettes are more expensive than normal tobacco:  *Yes*  *No*  *Do not know* | 21 (41.2)  14 (27.4)  16 (31.4) | 62 (30.9)  67 (33.3)  72 (35.8) | .37 |
| Electronic cigarettes are safer than tobacco:  *Yes*  *No*  *Do not know* | 22 (43.1)  24 (47.1)  5 (9.8) | 88 (44.0)  71 (35.5)  41 (20.5) | .14 |
| Electronic cigarettes are effective devices for smoking cessation:  *Yes*  *No*  *Do not know* | 13 (25.5)  33 (64.7)  5 (9.8) | 66 (33.0)  98 (49.0)  36 (18.0) | .12 |
| As a Public Health professional, would you recommend the electronic cigarette as smoking cessation aid to a patient?  *Yes*  *No*  *Do not know* | 8 (16.0)  34 (68.0)  8 (16.0) | 60 (44.8)  127 (63.2)  14 (29.8) | **.04** |
| As a Public Health professional, would you recommend the electronic cigarette to a patient for reducing the number of smoked cigarettes?  *Yes*  *No*  *Do not know* | 19 (37.3)  25 (49.0)  7 (13.7) | 90 (44.8)  93 (46.3)  18 (8.9) | .47 |
| Do you think that the concomitant use of electronic cigarettes and tobacco will effectively reduce the number of smoked cigarettes?  *Yes*  *No*  *Do not know* | 18 (35.3)  25 (49.0)  8 (15.7) | 73 (36.3)  90 (44.8)  38 (18.9) | .82 |
| Do you think that medical community and healthcare workers should take a position in favour of the electronic cigarettes?  *Yes*  *No*  *Do not know* | 14 (27.4)  24 (47.1)  13 (25.5) | 58 (29.0)  106 (53.0)  36 (18.0) | .48 |
| Do you think that electronic cigarettes should be prohibited?  *Yes*  *No*  *Do not know* | 13 (25.5)  29 (56.9)  9 (17.6) | 53 (26.4)  113 (56.2)  35 (17.4) | .99 |
| Have you heard of modified-risk tobacco?  *Yes*  *No* | 14 (27.5)  37 (72.5) | 48 (23.9)  153 (76.1) | .60 |
| The health risk of modified-risk tobacco products compared to smoking is:  *Higher*  *Equal*  *Lower*  *Do not know* | 0 (0.0)  6 (12.0)  7 (14.0)  37 (74.0) | 6 (3.0)  23 (11.6)  26 (13.1)  143 (72.2) | .85 |
| The health risk of modified-risk tobacco products compared to electronic cigarettes is:  *Higher*  *Equal*  *Lower*  *Do not know* | 9 (17.7)  3 (5.9)  2 (3.9)  37 (72.5) | 14 (7.1)  22 (11.1)  6 (3.0)  156 (78.8) | .10 |
| As a Public Health professional, would you recommend modified-risk tobacco products to reduce tobacco-related problems?  *Yes*  *No*  *Do not know* | 2 (3.9)  14 (27.4)  35 (68.6) | 12 (6.1)  45 (22.7)  141 (71.2) | .76 |
| ** Number for each item may not add up to total number of study population due to missing value* | | | |

**Table S11.** Participants’ responses on e-cigarettes and tobacco harm reduction. Differences in responses amongst MRPH by...

| **Questions *** | **By country**  **(n = 215*^§^*)** | **By year of residency**  **(n = 203)** | By setting of practice  (n = 226*^§^*) |
| --- | --- | --- | --- |
| The health risk of nicotine replacement therapies compared to smoking is:  *Higher*  *Equal*  *Lower*  *Do not know* | .009 | .44 | .17 |
| The health risk of electronic cigarettes compared to smoking is:  *Higher*  *Equal*  *Lower*  *Do not know* | .86 | .77 | .92 |
| The harmful effect of electronic cigarettes is due to the diethylene glycol:  *Yes*  *No*  *Do not know* | .15 | .88 | .91 |
| Electronic cigarettes can generate addiction:  *Yes*  *No*  *Do not know* | .26 | .71 | .12 |
| The dependence potential of electronic cigarettes compared to smoking is:  *Higher*  *Equal*  *Lower*  *Do not know* | .83 | .71 | .95 |
| Electronic cigarettes are more expensive than normal tobacco:  *Yes*  *No*  *Do not know* | .28 | .52 | .57 |
| Electronic cigarettes are safer than tobacco:  *Yes*  *No*  *Do not know* | .22 | .60 | .16 |
| Electronic cigarettes are effective devices for smoking cessation:  *Yes*  *No*  *Do not know* | .40 | .15 | .09 |
| As a Public Health professional, would you recommend the electronic cigarette as smoking cessation aid to a patient?  *Yes*  *No*  *Do not know* | .64 | .75 | .52 |
| As a Public Health professional, would you recommend the electronic cigarette to a patient for reducing the number of smoked cigarettes?  *Yes*  *No*  *Do not know* | .70 | .93 | .63 |
| Do you think that the concomitant use of electronic cigarettes and tobacco will effectively reduce the number of smoked cigarettes?  *Yes*  *No*  *Do not know* | .98 | .27 | .64 |
| Do you think that medical community and healthcare workers should take a position in favour of the electronic cigarettes?  *Yes*  *No*  *Do not know* | .13 | .92 | .63 |
| Do you think that electronic cigarettes should be prohibited?  *Yes*  *No*  *Do not know* | .71 | .98 | .69 |
| Have you heard of modified-risk tobacco?  *Yes*  *No* | .07 | .74 | .97 |
| The health risk of modified-risk tobacco products compared to smoking is:  *Higher*  *Equal*  *Lower*  *Do not know* | .09 | .79 | .74 |
| The health risk of modified-risk tobacco products compared to electronic cigarettes is:  *Higher*  *Equal*  *Lower*  *Do not know* | .09 | .84 | .40 |
| As a Public Health professional, would you recommend modified-risk tobacco products to reduce tobacco-related problems?  *Yes*  *No*  *Do not know* | .36 | .54 | .97 |
| *** Number for each item may not add up to total number of study population due to missing value  *^§^* Observations categorized as “*others*” were excluded from the analysis | | | |
